# Convergent Validity of the PUTS

**DOI:** 10.3389/fpsyt.2016.00051

**Published:** 2016-04-07

**Authors:** Valerie Cathérine Brandt, Christian Beck, Valeria Sajin, Silke Anders, Alexander Münchau

**Affiliations:** ^1^Department of Paediatric and Adult Movement Disorders and Neuropsychiatry, Center of Brain, Behavior and Metabolism, University of Lübeck, Lübeck, Germany; ^2^Department of Neurology, University of Lübeck, Lübeck, Germany; ^3^Department of Neurology, Institute of Social and Affective Neuroscience, University of Lübeck, Lübeck, Germany

**Keywords:** Tourette syndrome, tic, premonitory urge, PUTS, real-time urge monitor

## Abstract

Premonitory urges are a cardinal feature in Gilles de la Tourette syndrome. Severity of premonitory urges can be assessed with the “Premonitory Urge for Tic Disorders Scale” (PUTS). However, convergent validity of the measure has been difficult to assess due to the lack of other urge measures. We investigated the relationship between average real-time urge intensity assessed by an in-house developed real-time urge monitor (RUM), measuring urge intensity continuously for 5 min on a visual analog scale, and general urge intensity assessed by the PUTS in 22 adult Tourette patients (mean age 29.8 ± 10.3 SD, 19 males). Additionally, underlying factors of premonitory urges assessed by the PUTS were investigated in the adult sample using factor analysis and were replicated in 40 children and adolescents diagnosed with Tourette syndrome (mean age 12.05 ± 2.83 SD, 31 males). Cronbach’s α for the PUTS 10 was acceptable (α = 0.79) in the adult sample. Convergent validity between average real-time urge intensity scores (as assessed with the RUM) and the 10-item version of the PUTS (*r* = 0.64) and the 9-item version of the PUTS (*r* = 0.66) was good. A factor analysis including the 10 items of the PUTS and average real-time urge intensity scores revealed three factors. One factor included the average real-time urge intensity score and appeared to measure urge intensity, whereas the other two factors can be assumed to reflect the (sensory) quality of urges and subjective control, respectively. The factor structure of the 10 PUTS items alone was replicated in a sample of children and adolescents. The results indicate that convergent validity between the PUTS and the real-time urge assessment monitor is good. Furthermore, the results suggest that the PUTS might assess more than one dimension of urges, and it may be worthwhile developing different subscales of the PUTS assessing premonitory urges in terms of intensity and quality, as well as subjectively experienced control over tics and premonitory urges.

## Introduction

Premonitory urges, or simply “urges,” are aversive subjective sensations that have been described to precede tics in patients suffering from Gilles de la Tourette syndrome (GTS) (1, 2).

In contrast to entirely involuntary movements in other movement disorders, tics can be suppressed for limited time intervals. However, during tic suppression, unpleasant urges tend to increase until relieved by a tic (3–5). Therefore, tics are frequently experienced as voluntary responses to urges (4). Approximately 80–90% of GTS patients report to experience urges (1, 6, 7); hence, urges may play a key role in understanding GTS. Although the onset of urges appears to be delayed relative to tic onset by approximately 3 years, this finding might be due to difficulties assessing urges in 5- to 7-year olds (1, 7). Premonitory urges typically occur at the location where a tic is about to occur, but can also be experienced as a general inner tension (1). They can be experienced as “warm” or “cold,” “pressure-like,” or “tickling” sensations (8). In terms of intensity or urgency, premonitory urges have been likened to an itch (9).

A decade ago, Woods and Colleagues developed a short questionnaire to capture urge severity in children with tics (6). This questionnaire has been shown to have good psychometric properties (6, 10) and was later validated in adults (2, 11). The items of the Premonitory Urge for Tic Disorders Scale (PUTS) assess different sensory qualities of the urge, such as tickling, rising inner tension, or a “not just right” feeling. Moreover, questions cover the frequency of urge–tic associations, and the relief patients may experience after a tic has been executed.

Assessing convergent and discriminant validity of a new questionnaire are methods typically applied in psychometrics in order to ensure that the questionnaire measures the theoretical construct it was designed to measure and can discriminate between this construct and closely related constructs. However, investigating the convergent validity of the PUTS has been difficult because research concerning urges is relatively young, and there is a lack of instruments measuring a comparable construct. Furthermore, despite the wide use of the instrument, we are not aware of any study addressing the question of whether the PUTS assesses more than one dimension of urge phenomenology. Factor analyses are commonly applied when evaluating whether a questionnaire measures multiple dimensions of a construct or several constructs, especially when the questionnaire has several subscales (e.g., impulsivity, hyperactivity, and attention). The PUTS was designed as a one-dimensional questionnaire assessing urge severity, but studies showing that urge severity measured by the PUTS correlates positively with tic severity, obsessive–compulsive symptoms and anxiety (6, 10, 12, 13) suggest that the PUTS may reflect a multidimensional construct.

The current study uses a newly developed real-time urge monitor (RUM) to examine convergent validity between the PUTS and average tic-related urge intensity measured in real time. Furthermore, we assessed the discriminant validity between the PUTS and measures of attention-deficit hyperactivity disorder (ADHD) and obsessive–compulsive disorder (OCD). Moreover, the study examines whether the PUTS might measure more than one dimension of the urge phenomenon.

## Materials and Methods

### Participants – Clinical Assessment

Twenty-two patients (mean age 29.82 ± 10.34 SD, range = 17–55; 19 males) with a GTS diagnosis according to DSM-5 criteria (14) were included in this study. All patients gave their written informed consent prior to the study. Additionally, questionnaire data of 40 children and adolescents with a GTS diagnosis (mean age 12.05 ± 2.83 SD, range = 7–17; 31 males) were included in the study. Informed written assent was given by the children and written consent was given by their parents. The study was reviewed and approved by the local ethics committee and conformed to the Declaration of Helsinki.

Gilles de la Tourette syndrome symptom severity was assessed using the clinician-rated Yale Global Tic Severity Scale [YGTSS (15)]. In adults, symptoms of ADHD in childhood were rated on the German short version of the “Wender Utah Rating Scale” [WURS-K (16)], whereas current ADHD symptoms were assessed with the German ADHD self-rating scale [ADHD-SB (17)]. Symptoms of OCD were measured with the “Yale–Brown Obsessive–Compulsive Disorder Scale” [Y-BOCS (18)]. In children, symptoms of ADHD were assessed using the German parent-rated “FBB-ADHD” scale [“Diagnostik-System für Psychische Störungen nach ICD 10 und DSM-IV für Kinder und Jugendliche II,” DISYPS-II (19)] or the clinician-rated ADHD DSM-IV checklist [ADHD-DC (20)]. Symptoms of OCD were assessed with the “Yale–Brown Obsessive–Compulsive Disorder Scale for Children” [CY-BOCS (18, 21)].

### The PUTS Scale

Premonitory urges in general were measured using the validated German version of the PUTS (11). The PUTS is a 10-item self-rating scale and was originally developed to assess the intensity of urge phenomena on a 1–4 Likert rating scale (6). However, the last item has been removed from the PUTS score, because it was found to show small correlations with the rest of the scale (6, 10).

### Experimental Procedure of the Real-Time Urge Monitor

**Figure 1:**
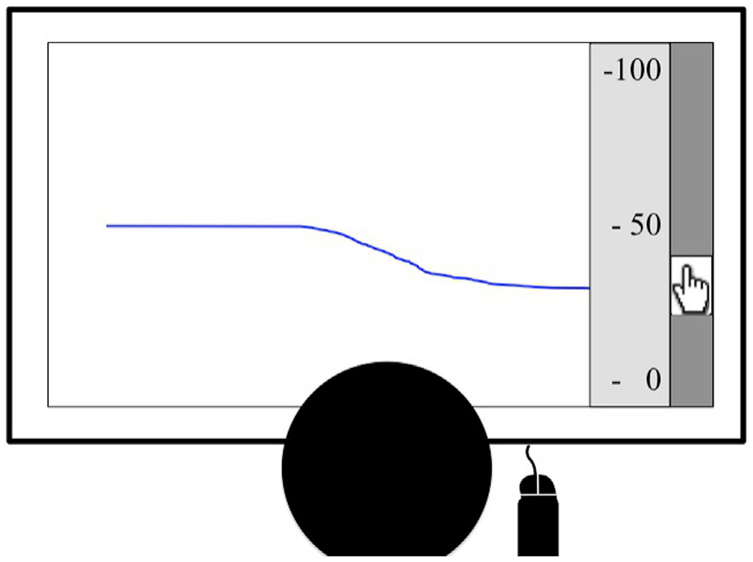
**The figure shows an example of the real-time urge monitor**. After a countdown, a blue line started moving across the screen continuously. Patients were asked to use the scroll bar on the right to continuously indicate the intensity of their current urge to tic on a scale from 0 to 100, displayed on the right of the screen.

Adult patients (*N* = 22) were seated in front of a Sony Vaio laptop (15″ screen) and were familiarized with the task. They were instructed to perform a continuous rating of their urge to tic over a period of 5 min. The right-hand side of the laptop screen showed a vertical intensity scale from 0 to 100, and patients were asked to indicate the intensity level of their current urge to tic, with 0 being no urge at all and 100 being the strongest urge intensity they typically experienced (see Figure 1). During the whole course of the experiment, patients were asked not to suppress any tics and to tic freely.

The task started when patients pressed a button. The button press initiated a countdown (3–2–1–0). At time 0, a blue line moved across the screen (at an intensity level 50), starting on the right-hand side of the screen, crossing the screen within 10 s. Patients were asked to continuously adjust the level of the blue line according to their subjectively experienced urge to tic. Hence, participants could see their urge ratings for the previous 10 s at any time. Data were sampled at 10 Hz. Patients were given the opportunity of a 1-min practice run and were asked to start the task after the experimenter left the room.

### Data Analysis

The continuous RUM resulted in 3000 data points per 5 min. The first 10 s (100 urge data points) were excluded in order to allow patients time to adjust the urge level on the screen to the correct level. The remaining 2900 data points were aggregated into a mean real-time urge intensity score.

In order to assess the internal consistency of the PUTS, an indicator of the reliability of a questionnaire, Cronbach’s α was calculated. A value of α > 0.80 is generally considered good, a value of α > 0.70 acceptable. Reliability was assessed in adults and children/adolescents separately.

Convergent validity (the degree to which two measures assessing the same construct are related) between the PUTS and the average real-time urge intensity monitor was assessed by correlating the mean real-time urge intensity scores of the adult sample (*N* = 22) with the PUTS 9 and the PUTS 10 score, respectively, using Pearson’s *r*. Discriminant validity (the degree to which a measure can discriminate between the construct it was designed to measure and the construct it was not designed to measure) between the PUTS and OCD/ADHD measures was assessed using Pearson’s *r*.

In order to assess the discriminatory power of individual PUTS items in the adult GTS sample, i.e., how well individual items of the PUTS capture the construct measured by the questionnaire overall, item-test correlations were performed between items and the PUTS 9 score as well as the PUTS 10 score (part-whole corrected) using Pearson’s *r*. Additionally, the individual PUTS items were correlated with the average real-time urge intensity score in order to investigate which PUTS items best captured urge intensity.

Thereafter, two factor analyses were run. The first included only the 10 items of the PUTS, in order to assess whether the PUTS might reflect more than one dimension of premonitory urges. The second additionally included the real-time urge intensity score as an item, in order to determine which one of the factors might best represent the construct of urge intensity. Finally, a factor analysis including only the 10 items of the PUTS was computed in the young sample (*N* = 40) to assess whether the factor structure was similar in a younger, independent sample.

## Results

### Clinical Assessment

In the adults sample, mean total tic severity according to the YGTSS (0–50) was 17.05 ± 7.7 SD, and the mean PUTS 9 score (10–36) was 21.05 ± 5.78 SD. Results from the Y-BOCS showed that none of the patients exceeded the overall cut off for clinically relevant OCD symptoms (16), with values ranging from 0 to 14 (3.19 ± 4.85). However, WURS-K values ranged from 0 to 48 (15.98 ± 13.65) indicating that 4 patients scored in the clinical range (cut off = 30); three of these patients also fulfilled DSM-5 criteria for ADHD (14).

In the young sample, mean total tic severity according to the YGTSS50 was 17.77 ± 8.12 SD. The mean PUTS 9 score was 17.83 ± 6.38 SD. Mean ADHD values according to the FBB-ADHD (*N* = 25) scale were 0.96 ± 0.77 SD and according to the ADHD-DC (*N* = 13) scale were 0.15 ± 0.14 SD. Nine out of the 40 children scored in the clinical range and/or had an ADHD diagnosis according to DSM-5 (14). The mean CY-BOCS score (*N* = 36) was 3.03 ± 6.3 SD; five of these patients had a diagnosis in the OCD spectrum.

### Internal Consistency of the PUTS

Cronbach’s α across the 10 items of the PUTS was acceptable in the adult sample (α = 0.79) and good in the young sample (α = 0.84).

Item–test correlations between individual PUTS items and the PUTS 9/PUTS 10 score showed that items 1, 6, and 9 consistently did not assess the overall construct of the PUTS as well as the other items (please see Table 1 for coefficients; for items, see Table 2). As previously found, item 10 also showed a very small correlation with the overall test score (*r* = −0.02). Excluding items 1, 6, 9, and 10 increased internal reliability of the PUTS in the adult sample (α = 0.84), but not the young sample (α = 0.84).

**Table 1:** **Correlations between PUTS items and RUM/ADHD/OCD measures**.

PUTS	RUM	PUTS 9	PUTS 10	Y-BOCS	WURS-K	ADHD-SB	ADHD-A	ADHD-H	ADHD-I
*Item 1*	**0.13**	0.25	0.28	**−**0.18	**−**0.35	**−**0.01	**−**0.14	**−**0.09	0.17
*Item 2*	0.55**	0.67**	0.68**	0.25	0.29	0.13	0.002	0.34	**−**0.01
*Item 3*	**0.36**	0.58**	0.6**	0.59**	0.56**	0.44*	0.26	0.64**	0.27
*Item 4*	0.51*	0.8**	0.79**	0.53*	0.46*	0.27	0.26	0.26	0.15
*Item 5*	**0.35**	0.72**	0.75**	0.43*	0.27	0.18	0.22	0.22	0.18
*Item 6*	0.55**	0.3	0.22	0.08	0.32	0.25	0.24	0.27	0.08
*Item 7*	0.6**	0.58**	0.55**	0.09	0.02	0.02	**−**0.09	0	33
*Item 8*	0.67**	0.47*	0.41	**−**0.11	0.001	0.1	**−**0.13	0.23	0.35
*Item 9*	**−0.01**	0.21	0.26	0.5*	0.19	0.09	0.18	**−**0.11	0.18
*Item 10*	**−0.17**		**−**0.02	0.16	**−**0.23	**−**0.32	**−**0.27	**−**0.25	**−**0.34
***Multitrait-multimethod matrix***
*RUM*	1								
*PUTS 9*	0.66**	1							
*PUTS 10*	0.64**	0.99	1						
*Y-BOCS*	0.11	0.41	0.43*	1					
*WURS-k*	0.12	0.35	0.32	0.51*	1				
*ADHD-SB*	0.2	0.25	0.21	0.34	0.61**	1			
*ADHSD-A*	**−**0.01	0.15	0.11	0.38	0.57**	0.89**	1		
*ADHD-H*	0.26	0.35	0.31	0.28	0.73**	0.83**	0.54*	1	
*ADHD-I*	0.05	0.3	0.25	0.09	0.58**	0.82**	0.61**	0.67**	1

*RUM, real-time urge monitor; PUTS, Premonitory Urge for Tic Disorders Scale; Y-BOCS, Yale–Brown Obsessive–Compulsive Scale; WURS-K, Wender Utah Rating Scale Short Form; ADHD-SB, Attention Deficit Hyperactivity Disorder Self-Rating Scale; ADHD-A, inattention subscale of the ADHD-SB; ADHD-H, hyperactivity subscale of the ADHD-SB; ADHD-I, impulsivity subscale of the ADHD-SB*.

*p < 0.05.

**p < 0.01.

**Table 2:** **Rotated factor analysis of the PUTS and the real-time urge measure**.

Items	Adult sample			Young sample		
	F1	F2	F3	F1	F2	F3
*Item 1:* Right before I do a tic I feel like my insides are itchy	0.02	**0.60**	0.59	**0.67**	**−**0.05	0.09
*Item 2:* Right before I do a tic I feel pressure inside my brain or body	**0.72**	0.32	0.06	**0.76**	0.4	0.04
*Item 3:* Right before I do a tic I feel “wound up” or tense inside	**0.85**	0.01	**−**0.05	0.34	**0.82**	**−**0.03
*Item 4:* Right before I do a tic I feel like something is not “just right”	**0.86**	0.27	0.03	0.07	**0.82**	0.28
*Item 5:* Right before I do a tic I feel like something is not complete	**0.82**	0.18	0.19	0.06	**0.87**	0.17
*Item 6:* Right before I do a tic I feel like there is energy in my body that needs to get out	0.44	0.18	**−0.76**	0.22	0.09	**0.66**
*Item 7:* I have these feelings almost all the time before I do a tic	0.27	**0.82**	**−**0.02	**0.75**	0.22	0.5
*Item 8:* These feelings happen for every tic I have	0.14	**0.90**	**−**0.26	**0.83**	0.1	0.16
*Item 9:* After I do the tic, the itchiness, energy, pressure, tense feelings, or feelings that something is not “just right,” or complete go away, at least for a while	0.38	**−**0.004	**0.62**	0.28	0.07	**0.86**
*Item 10:* I am able to stop my tics even if only for a short period of time	0.14	**−**0.22	**0.76**	**−**0.11	0.39	**0.54**
*Real-time urge intensity score*	0.46	**0.63**	**−**0.32			

*F, factor; PUTS, Premonitory Urge for Tic Disorders Scale*.

### Convergent Validity between the PUTS and Real-Time Urge Intensity

The average real-time urge intensity score was highly correlated with the PUTS 10 (*r* = 0.64, *p* = 0.001) and the PUTS 9 (*r* = 0.66, *p* = 0.001). PUTS items 1, 9, and 10 showed weak and non-significant correlations with the average real-time urge intensity score (*r* < 0.2, *p* > 0.4; for a full list of correlations between real-time urge intensity score and single items of the PUTS, please see Table 1). Excluding these items increased the overall correlation between the mean real-time urge intensity score and the PUTS 10 (*r* = 0.71, *p* < 0.001).

The YGTSS motor tic severity score showed medium correlations with the PUTS (PUTS 10: rho = 0.43, *p* = 0.048; PUTS 9: rho = 0.48, *p* = 0.025) and a medium non-significant correlation with real-time urge intensity (rho = 0.37, *p* = 0.09). The number of tics per 5 min (121.36 ± 60.56) correlated significantly with real-time urge intensity (*r* = 0.46, *p* = 0.03), but not with the PUTS 9 (*r* = 0.36, *p* = 0.103) or PUTS 10 score (*r* = 0.39, *p* = 0.073).

### Discriminant Validity between Urge Measures and ADHD/OCD Measures

There was a significant correlation between the PUTS 10 and the Y-BOCS (see Table 1). Correlations between single items of the PUTS and clinical scores showed that the Y-BOCS was significantly correlated with items 3 (*r* = 0.59, *p* = 0.004), 4 (*r* = 0.53, *p* = 0.012), 5 (*r* = 0.43, *p* = 0.047), and 9 (*r* = 0.5, *p* = 0.019; Table 1). The WURS-K score correlated significantly with items 3 (*r* = 0.56, *p* = 0.006) and 4 (*r* = 0.46, *p* = 0.032), ADHD-SB also correlated significantly with item 3 (*r* = 0.44, *p* = 0.047), especially with hyperactivity (*r* = 0.64, *p* = 0.002). However, the WURS-K and the Y-BOCS total scores were also significantly correlated (*r* = 0.51, *p* = 0.014; Table 1).

There were no significant correlations between the real-time urge intensity score and ADHD/OCD scores (Table 1).

### Dimensions of the PUTS

A factor analysis with Varimax rotation across the PUTS 10 and the real-time urge intensity score revealed three factors. Items 2, 3, 4, and 5 loaded highest on the first factor, whereas the real-time urge intensity score loaded highest on the second factor together with items 1, 7, and 8. Item 1 did not load clearly on one factor though but was almost equally distributed between factors 2 and 3. Items 6, 9, and 10 loaded highest on the third factor (Table 2). The same structure emerged when only the 10 PUTS items were included in the analysis.

A very similar structure of the 10 PUTS items was found in the young sample. The only item that differed was Item 2, loading highest on the intensity factor instead of the quality factor (see Table 2).

## Discussion

### Construct Validity of the PUTS

The current study sought to assess the convergent validity of the PUTS with a measure that assesses urge intensity in real time. Average real-time urge intensity correlated highly with the PUTS 9 and PUTS 10 scores. This shows good convergent validity of the PUTS with a measure that tracks urge intensity over a limited time interval. However, low correlations between urge intensity assessed by the real-time urge intensity monitor and individual items (1, 9, and 10) of the PUTS suggest that not all items of the PUTS tap into the construct of urge intensity. These findings were reflected by the results regarding internal consistency of the PUTS.

Internal consistency of the PUTS 10 was acceptable (α = 0.79) in adult GTS patients, replicating previous findings (2, 6). However, consistency could be increased by excluding a number of items that showed small to medium correlations with the overall construct assessed by the PUTS. Item 10, referring to the ability to stop one’s tics, was already removed from the PUTS total score in most recent studies (12, 22). Item 1, assessing an “itch-like” urge quality, item 6, characterizing urges as an energy that needs to get out, and item 9, assessing to what degree urges subside after tics, also appeared to assess a different construct than urge intensity. Excluding items 1, 6, 9, and 10 increased internal consistency and convergent validity in the adult sample. However, instead of excluding these items, it might be worthwhile investigating and building on the different underlying dimensions of urges that the PUTS might assess. Factor analyses including all 10 items of the PUTS (with and without real-time urge intensity score) revealed a three-factor solution.

Items loading on the first factor assess whether patients feel “a pressure,” “wound up,” “like something is not ‘just right’ or ‘incomplete’” and might be interpreted to assess the *quality* of premonitory sensations. The second factor included the average real-time urge intensity score and two items assessing in how far patients had these “feelings almost all the time” before a tic and “for every tic” and might reflect the overall *intensity* of premonitory urges. Item 1 (“…*my insides are itchy*”) also loaded highest on the second factor. However, it loaded almost equally high on factor three (0.60 vs. 0.59) and might not clearly reflect any of the underlying dimensions. Surprisingly, it was not included in the first factor, assessing quality of urges.

The third factor comprised item 9, assessing how much tics are associated with a relief in urges and item 10, referring to the patients ability to stop their tics. Additionally, item 6 assesses to what degree patients feel that there is “an energy” in their body that needs to get out before the tic and loaded highly negatively on factor three. The nature of these items suggests that the underlying factor may be related to the perceived *control* over tics and urges.

This pattern was largely replicated in 40 children and adolescents with GTS. In this sample, item 2, referring to urges as a pressure moved from the *quality* factor to the *intensity* factor.

### Convergent and Discriminant Validity

The medium correlation between the overall PUTS score and the motor tic severity score of the YGTSS suggests that both questionnaires assess distinct, but related constructs. This cannot strictly be taken as proof of validity of the PUTS because the YGTSS does not aim to assess the same construct as the PUTS. Moreover, previous studies regarding the association between the PUTS and the YGTSS have rendered mixed results (2, 6, 10, 12, 23, 24). This suggests that the relationship between urge severity and tic severity either depends on the sample characteristics (e.g., comorbidities) or that they are not always sufficiently captured by the PUTS and/or the YGTSS to reveal their relationship.

A significant correlation in the medium range between the number of tics assessed in real time and urge intensity assessed in real time supports the notion that tic severity and urge severity are related, but distinct phenomena, independent of the measure used to assess them. The finding that correlations across different measures (real-time urge intensity with YGTSS motor tic severity; real-time number of tics with PUTS scores) were lower and non-significant could be due to the different time windows assessed by questionnaires and real-time instruments. Questionnaires aim to assess phenomena in general, whereas the RUM assesses severity of tics and urges in a small time window. Tics wax and wane and urge severity assessed at a particular point in time can differ from urge severity judged over a longer time period and averaged across all tics that the patient recalls while filling out the questionnaire.

The PUTS 10, but not the PUTS 9 score, correlated significantly with the Y-BOCS, but not with ADHD measures, replicating previous mixed findings on the association between symptoms of OCD or ADHD and urges measured by the PUTS (6, 10, 12, 13, 24, 25). However, we would not classify significant correlations with the Y-BOCS as convergent validity because the questionnaires aim to assess very different constructs. On the contrary, it might be more useful if items assessing urge intensity associated with tics did not tap into related phenomena that might be associated with obsessions or compulsions. Hence, the discriminant validity of the PUTS was not good because it did not clearly measure urge intensity only associated with tics.

The majority of OCD patients with premonitory sensations experience “just-right” sensations (26), whereas the majority of GTS patients describe it as an impulse or urge to move (4). Based on the correlational pattern between single items of the PUTS and measures of OCD and ADHD, it appears likely that specific items, related to the *quality* of urges, tap into phenomena that are typically associated with OCD (i.e., “not just right” feelings or feelings of incompleteness) or ADHD (i.e., feeling “wound up”) and not specifically with the urge to tic. Similar associations between PUTS items and OCD symptoms have previously been found (10, 13).

Urge intensity *per se* might not be associated with symptoms of OCD and ADHD. In line with this assumption, items loading on the *intensity* factor of the PUTS and the real-time urge intensity score were not significantly correlated with OCD or ADHD scores, suggesting that urge *intensity* is independent from comorbidities.

### Limitations and Future Directions

The main limitation of the study is the sample size. Although this should not be problematic for the results concerning convergent validity between the PUTS and the real-time urge measure, more patients will be required to draw firm conclusions concerning the underlying dimensions assessed by the PUTS. Despite the replication of a very similar three-factor solution in the young sample, it might be useful for researchers to pool their PUTS data and investigate whether these dimensions can be replicated in large samples of at least 50 individuals (27).

If the structure can be replicated, the PUTS might be further developed into several subscales with more items on each scale. The subscale assessing urge intensity should then have high discriminant validity, purely assessing urge intensity regarding tics and not tap into phenomena that might also be associated with comorbidities. The subscales assessing quality of urges and perceived control over urges/tics might be very interesting and useful with regard to comorbidities. For instance, individuals with higher ADHD scores were less likely to say that they could stop their tics in this sample, whereas patients with higher OCD scores were more likely to say that urges subsided after tics. Although correlations with ADHD symptoms were not significant, perceived control might be an interesting question to pursue in the future. Furthermore, intelligence has been shown to be associated with some executive functions (28), and future studies might evaluate the role of intelligence in perceived and actual tic control. Until now, research investigating differences in premonitory urges has mostly focused on the different qualities of the experienced sensation (e.g., just right feeling, impulse, and energy release), and future studies might investigate the underlying dimensions of urges more comprehensively.

## Author Contributions

VB: conceptualization/design, data acquisition, analysis, interpretation, draft, approval of the manuscript for publication, and agrees to be accountable for all aspects of the work. CB: conceptualization/design, programming of the task, critical revision for important intellectual content, approval of the manuscript for publication, and agrees to be accountable for all aspects of the work. VS: data acquisition, analysis, critical revision for important intellectual content, approval of the manuscript for publication, and agrees to be accountable for all aspects of the work. SA: conceptualization/design, critical revision for important intellectual content, approval of the manuscript for publication, and agrees to be accountable for all aspects of the work. AM: conceptualization/design, critical revision for important intellectual content, approval of the manuscript for publication, and agrees to be accountable for all aspects of the work.

## Conflict of Interest Statement

The authors declare that the research was conducted in the absence of any commercial or financial relationships that could be construed as a potential conflict of interest.
